# Quality of vision clinical outcomes for a new fully-refractive extended depth of focus Intraocular Lens

**DOI:** 10.1038/s41433-024-03039-8

**Published:** 2024-04-05

**Authors:** Dean Corbett, Daniel Black, Timothy V. Roberts, Brendan Cronin, David Gunn, Chandra Bala, Patrick Versace, Linda Tsai, Eleni Papadatou, Aixa Alarcon, Srividhya Vilupuru

**Affiliations:** 1 Auckland Eye, Auckland, New Zealand; 2 Sunshine Eye Clinic, Birtinya, QLD Australia; 3 Vision Eye Institute, Sydney, NSW Australia; 4 The University of Sydney, Sydney Medical School, Faculty of Medicine and Health, Sydney, NSW Australia; 5 Queensland Eye Institute Foundation, South Brisbane, QLD Australia; 6 personalEYES, Parramatta, NSW Australia; 7 Dr Versace, SMS healthcare, University of New South Wales, Sydney, NSW Australia; 8 Johnson and Johnson MedTech, Irvine, CA USA; 9 Johnson and Johnson MedTech, Groningen, The Netherlands

**Keywords:** Outcomes research, Lens diseases

## Abstract

**Background/objective:**

To evaluate the visual performance of a purely refractive extended depth of focus (EDF) intraocular lens (IOL).

**Subjects/methods:**

A prospective, multi-center, randomized, subject/evaluator-masked study. Subjects were bilaterally implanted with the EDF test (Model ZEN00V, TECNIS PureSee™ IOL, *N* = 60) or an enhanced monofocal control (Model ICB00, TECNIS Eyhance™ IOL, *N* = 57) IOL. Monocular corrected distance (CDVA), intermediate (DCIVA), near acuities (DCNVA) and patient reported visual symptoms were evaluated at the 6-month visit. Monocular mesopic contrast sensitivity (CS) and depth of focus (DOF) testing were assessed at 3 months.

**Results:**

CDVA (Mean ± SD) was −0.06 ± 0.08 for test and −0.05 ± 0.08 logMAR for control groups. DCIVA was 0.13 ± 0.08 for test and 0.18 ± 0.14 logMAR for control groups (*p* = 0.0127). DCNVA was 0.37 ± 0.10 for test and 0.43 ± 0.16 logMAR for control groups (*p* = 0.0137). Test lens was statistically superior for intermediate and near. Overall, 91.7% (halos), 95.0% (starbursts) and 95.0% (glare) of test lens patients reported that they did not experience, were not bothered, or were slightly bothered by specific visual symptoms, compared to 98.2%, 100% and 96.5% in the control group. The DOF range over which monocular visual acuity was 0.20 logMAR or better was −1.6 D for the test lens. Mesopic CS was comparable between both groups, falling within 0.11 log units for all measured cycles per degree with and without glare.

**Conclusion:**

The EDF IOL demonstrated extended range of vision and statistically superior intermediate and near performance compared to the monofocal IOL. Distance visual acuity, contrast sensitivity and dysphotopsia profile were similar to the monofocal IOL.

## Introduction

Monofocal intraocular lenses (IOLs) are used globally for visual correction after cataract removal. Standard monofocal IOLs have a single focal point and are typically calculated to be distance-dominant, resulting in excellent distance vision while requiring spectacle correction for reading and intermediate tasks [1, 2]. Enhanced monofocal IOLs can slightly extend the range of vision to provide functional intermediate vision while maintaining distance vision and a dysphotopsia profile similar to that of standard monofocal IOLs [3]. Multifocal IOLs are designed to increase spectacle independence by providing multiple distinct focal points in vision, from distance to intermediate and/or near [4]. However, the improvement in unaided near vision and increased spectacle independence with multifocal IOLs needs to be weighed against the patient experience of glare and halos and impact on contrast sensitivity and intermediate vision [2, 4–7].

EDF IOLs bridge the gap between monofocal and multifocal IOLs by elongating the focal point(s) to provide continuous, high-quality enhanced range of vision [6, 8, 9]. Optical designs include diffractive (TECNIS Symfony, Johnson and Johnson Surgical Vision, Irvine, CA, USA [8]), spherical aberration based (Mini WELL Ready IOL, SIFI S.p.A, Catania, Italy [10] and LuxSmar, Bausch & Lomb GmbH, Berlin, Germany [11]), pinhole effect (IC-8, AcuFocus Inc., Irvine, CA, USA [12, 13]), and newer “non-diffractive” technology (Vivity IOL, Alcon Laboratories, Fort Worth, TX, USA [14]). For example, TECNIS Symfony IOL was the first EDF lens, which provides good distance vision with improved intermediate and near vision compared to a standard monofocal IOL, and less dysphotopsia and superior contrast sensitivity compared to some multifocal IOLs [8, 15].

Despite the advantages of these technologies, reduced contrast sensitivity and/or more prevalent or bothersome dysphotopsias compared with monofocal IOLs may still be a factor for dissatisfaction in patients [9, 14, 16]. A need exists for an IOL option that can improve intermediate and near vision over standard monofocal IOLs while maintaining a high quality of vision and low levels of dysphotopsia.

The TECNIS PureSee IOL (Johnson and Johnson Surgical Vision, Irvine, CA, USA) is a next-generation EDF lens with a purely refractive design. The lens is designed to provide an increased range of vision for intermediate and near tasks while having distance vision, contrast sensitivity and dysphotopsia profile comparable to a monofocal IOL. The purpose of this study was to evaluate the visual performance and safety of the PureSee IOL (Model ZEN00V) compared to an enhanced monofocal in patients bilaterally implanted after cataract extraction.

## Methods

### Study design

This was a prospective, bilateral, randomized, subject and evaluator-masked comparative study conducted in Australia and New Zealand (ClinicalTrials.gov; NCT04890249). Data collected from a total of six study sites are included in the analysis. All patients provided written informed consent, and Independent Ethics Committee approval (Bellberry Limited, Human Research Ethics Committee and Health and Disability Ethics Committee) was obtained. The study was conducted in accordance with Good Clinical Practices, ISO14155:2011, the tenets of Declaration of Helsinki, and all other applicable laws and regulations of the countries in which the study was conducted.

### Inclusion and exclusion criteria

Patients were eligible for study inclusion if they were 22 years or older and scheduled to have bilateral cataract surgery, preoperative corrected distance visual acuity (CDVA) of 20/40 or worse with or without glare and/or experience significant cataract related visual symptoms, potential to achieve postoperative CDVA of 20/30 or better, normal corneal topography, predicted postoperative corneal astigmatism of less than 1.0 dioptre (D), and clear intraocular media other than cataract. Patients requiring an IOL power outside the range of +14.0 D to +26.0, pupil abnormalities, recent ocular trauma or surgery, ocular anomalies affecting postoperative outcomes were excluded from the study.

### IOL device description

The next generation refractive EDF TECNIS PureSee IOL, Model ZEN00V (test) was compared to an enhanced monofocal: the TECNIS Eyhance IOL, Model ICB00 (control). PureSee is a purely refractive, 1-piece soft acrylic aspheric foldable posterior chamber IOL designed for placement in the capsular bag. This IOL has the same overall geometry/dimensions as the control IOL and is made from SENSAR UV^2^ (OptiBlue) material. The TECNIS PureSee IOL has an anterior aspheric surface designed to compensate for average corneal spherical aberration and a posterior refractive surface designed to create a continuous change in power to extend the depth of focus.

The enhanced monofocal TECNIS Eyhance IOL is a 1-piece refractive foldable posterior chamber IOL made of SENSAR material that has a higher-order aspheric anterior surface design, with a continuous change in power from the periphery to the center to slightly extend the depth of focus [17].

### Randomization and surgical procedure

Eligible patients were enrolled and randomized (1:1) via electronic data capture system to undergo bilateral implantation with either the test or control IOLs. Prior to randomization, surgeons selected which eye to operate on first for each patient based on their standard clinical practice (e.g., the eye with worse cataract and poorer best-corrected distance vision). Surgery for the second eye was performed after the 1-week postoperative examination but not more than 30 days after the first-eye surgery. Surgeons employed their typical small-incision technique for cataract extraction, inserting the IOLs into the capsular bag using one of the validated insertion systems. All surgical outcomes were managed by the investigators to ensure minimum total postoperative refractive astigmatism of <1.0 D through incision type and placement. No additional refractive procedures were performed during the study period.

### Endpoints and assessments

Evaluators of vision testing and refractions, and all subjects, remained masked to the implanted IOL throughout the duration of the study.

#### Visual outcomes and quality of vision

At the 6-month postoperative visit visual acuities were measured using the Early Treatment Diabetic Retinopathy Study (ETDRS) chart on the Clinical Trial Suite (CTS, M&S Technologies, Inc.) under photopic lighting conditions (85 cd/m^2^). Manifest refractions were conducted at 4 m employing standard refractive technique and adjusted for optical infinity (−0.25 D of sphere).

Monocular visual acuity endpoints at 6 months included mean corrected distance VA (CDVA) at 4 m, distance corrected intermediate VA (DCIVA) at 66 cm, and distance corrected near VA (DCNVA) at 40 cm.

Monocular, corrected distance contrast sensitivity was measured in first eyes at the 3-month visit under mesopic lighting conditions (3 cd/m^2^) both with and without glare. This was measured using the CTS system and sinewave grating charts encompassing frequencies of 1.5, 3, 6, and 12 cycles per degree (cpd) at 2.5 m; a refraction adjustment was used. Monocular corrected distance defocus curve visual acuity testing was performed in first eyes only at the 3-month visit. Testing was conducted from +1.00 D through −2.50 D of defocus.

Visual symptoms, were documented noting their frequency, degree of impact (how bothersome) and difficulty resulting from their presence using the Patient Reported Visual Symptoms Questionnaire (PRVSQ) at the 6-month visit.

#### Subgroup assessment on the impact of refraction technique on range of vision

ZEN00V (*n* = 13) subjects from a single center returned for a prospective, one visit assessment. One eye of each subject was tested. The subjects had to have completed their 1-month postoperative visit for both eyes. The purpose was to determine the influence of the refractive technique on range of vision measured using defocus curve testing. A single optometrist conducted maximum plus for maximum visual acuity refraction (MPMVA) using the fogging technique. Monocular distance-corrected defocus curve (range from +1.00 D through −2.50 D) was measured at 4 m with the MPMVA technique.

#### Historical data from a standard monofocal IOL

The American National Standards Institute (ANSI) lists criteria for categorizing an EDF IOL in comparison to a standard monofocal IOL [18]. Historical visual acuity and visual symptoms data on standard monofocal IOL (Model ZCB00 [*n* = 131]) at 6 months are presented for comparison purposes. These data were collected using the same standardized methods as detailed in this paper and the data is published in the FDA Summary of Safety and Effectiveness Document [19]. The inclusion of the standard monofocal broadens the comparative framework, enhancing the understanding of the effectiveness and safety of the ZEN00V IOL as an EDF IOL.

### Statistical analysis

For monocular DCIVA and DCNVA, with 55 subjects in each lens group, there is 90% power to detect a 0.10 logMAR or greater difference in mean visual acuity between the ZEN00V and ICB00 lens groups using a two-sample *t* test at a one-sided alpha of 0.025 and assuming a standard deviation of 0.16 logMAR. For monocular CDVA, with 55 subjects in each lens group, there is 99% power to conclude non-inferiority in visual acuity between the ZEN00V and ICB00 lens groups at one-sided alpha of 0.05 with non-inferiority margin of 0.10 logMAR, assuming no difference between the IOLs and a standard deviation of 0.12 logMAR. At the end of the study, there were more than 55 subjects in each lens group; therefore, the sample size for this analysis is considered sufficiently powered.

All endpoints were evaluated in the safety population (i.e., no data imputation). Summary statistics included mean and standard deviation (±SD) for continuous variables. For categorical data, the frequency and proportion were computed. Monocular first eye CDVA was evaluated using a noninferiority approach with a margin of 1 line and one-sided alpha of 0.05. Analysis of monocular best corrected DCIVA and DCNVA was based on first eyes using one sided, two-sample *t*-tests with one-sided alpha level of 0.025. Frequency counts and proportions of first eyes achieving DCIVA of 0.20 logMAR (20/32 Snellen equivalent) were reported by IOL group.

The mean visual acuity and standard error (SE) at each dioptre of defocus was plotted for the range of the testing conducted. The depth of focus was estimated as the dioptric range between zero defocus and the point in the negative defocus curve that crosses the 0.20 logMAR threshold. Patient-Reported Visual Symptoms Questionnaire data were reported for subjects who have received the same test IOLs or same control IOLs in both eyes; data were tabulated with the frequency and proportion for each response by IOL group. All statistical analysis was conducted using SAS program (Version 9.4).

## Results

Between June 2021 and August 2022, a total of 120 subjects underwent implantation with ZEN00V (*n* = 62) or ICB00 (*n* = 58) IOL in at least one eye at six study sites. Among them, 62 ZEN00V and 57 ICB00 subjects were bilaterally implanted. 60 ZEN00V and 58 ICB00 subjects completed the final 6 months visit. Despite the on-going COVID-19 pandemic, patient accountability for first eyes in the ZEN00V group was 90.3% (56/62) and 96.8% (60/62) at 3 months and 6 months, respectively, while in the ICB00 group, it was 93.1% (54/58) and 100% (58/58) at 3 months and 6 months, respectively. Patient demographics were similar between the two IOL groups, exhibiting no significant differences (Table 1). The mean (± SD) age was 68.2 ± 8.5 and 69.8 ± 7.7 years in the ZEN00V and ICB00 IOL groups, respectively, and there were more females in both IOL groups. Monocular outcomes are reported for first implanted eyes only.

**Table 1:** Patient demographics observed during the study period.

Parameter	ZEN00V *N* = 62	ICB00 *N* = 58
Age (y), mean ± SD	68.2 ± 8.5	69.8 ± 7.7
Age Group (y), *n* (%)		
<60	8 (12.9)	4 (6.9)
60−69	22 (35.5)	26 (44.8)
70–79	29 (46.8)	22 (37.9)
≥80	3 (4.8)	6 (10.3)
Sex *n* (%)		
Male	25 (40.3)	19 (32.8)
Female	37 (59.7)	39 (67.2)
Race, *n* (%)		
Asian (including Indian)	10 (16.1)	9 (15.5)
Black	0	0
Native Hawaiian/Pacific Islander	0	0
Caucasian	49 (79)	47 (81)
Other	3 (4.8)	2 (3.4)
Ethnicity, *n* (%)		
Hispanic/Latino	0	1 (1.7)
Not Hispanic/Latino	62 (100%)	57 (98.3)
Iris Color, n (%)		
Blue/Gray	21 (33.9)	17 (29.3)
Brown/Black	20 (32.3)	16 (27.6)
Green/Hazel	21 (33.9)	25 (43.1)

### Quality of vision

#### Monocular distance corrected visual acuity

The mean (±SD) monocular photopic CDVA at 4 m was −0.06 ± 0.08 logMAR (Snellen 20/15) in the ZEN00V group compared with −0.05 ± 0.08 logMAR (Snellen 20/20) in the ICB00 group. The lower 2-sided 90% CI of the mean difference was −0.01 logMAR between ZEN00V and ICB00 which was better than the noninferiority margin of −0.1 logMAR, confirming that the next generation EDF was non inferior to the enhanced monofocal IOL. In comparison, the ZCB00 group showed −0.05 ± 0.09 logMAR (Snellen 20/20). Additionally, 100% of ZEN00V first eyes achieved BCDVA of 0.20 logMAR (Snellen 20/32) or better at 6 months.

#### Visual symptoms

Direct responses from the validated PRVSQ were used to assess the frequency, bother and difficulty, of visual symptoms in the past 7 days, at the 6-month visit.

With respect to frequency, in the ZEN00V group, 88.3% (53/60), 96.7% (58/60) and 100.0% (60/60) of patients reported that they never, rarely or sometimes experienced halos, starbursts and glare, respectively compared to 91.3% (52/57), 94.7% (55/57), 100% (57/57) in the ICB00 group and 95.4% (125/131), 96.2% (126/131) and 98.5% (129/131) in the historical ZCB00 group.

**Fig. 1:**
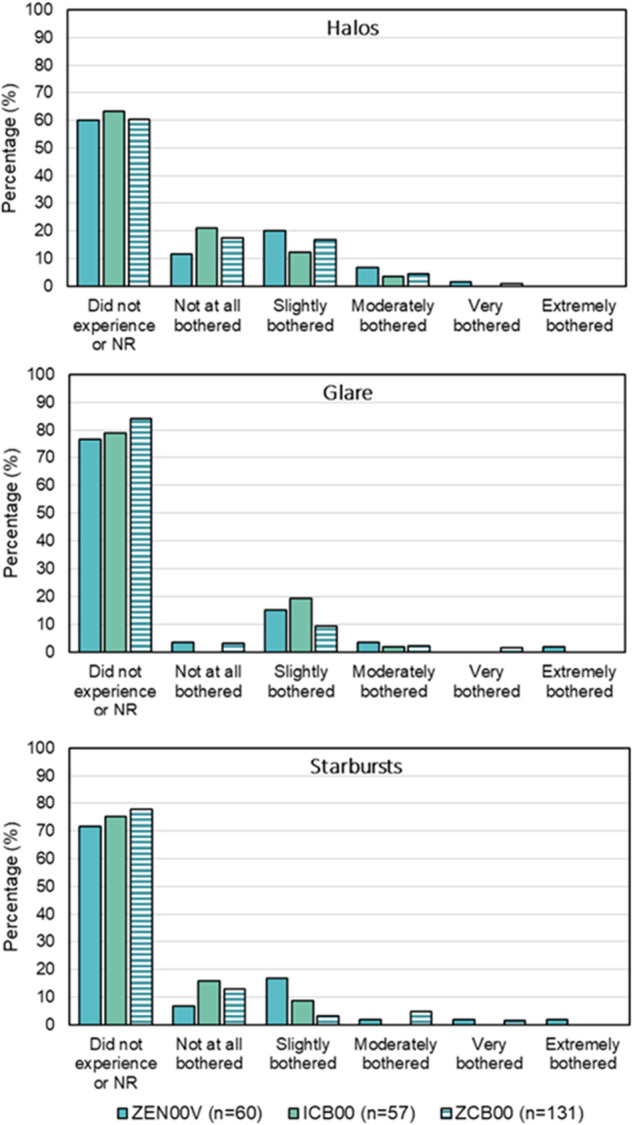
Level of bothersome (%) for ocular visual symptoms, including halos (top graph), glare (middle graph), and starbursts (bottom graph), acquired with PRVSQ at 6 months for ZEN00V, ICB00, and ZCB00 (historical control) bilaterally implanted patients. ‘NR’ indicates no response.

Figure 1 demonstrates low levels of bothersome symptoms in the ZEN00V, ICB00, and historical ZCB00 groups. The proportion of patients who did not experience, were not bothered, or were slightly bothered by visual symptoms were comparable between the ZEN00V group and the ICB00 group for halos (91.7% [55/60] vs 96.5% [55/57], respectively), starbursts (95.0% [57/60] vs 100% [57/57]), and glare (95.0% [57/60] vs 98.2% [56/57]). The ZCB00 group also reported similar results (halos: 94.7% [126/131]; starburst: 93.9% [123/131]; glare: 96.2% [126/131]).

In the ZEN00V group, 96.7% (58/60) of patients reported no difficulty with or did not experience halos, starbursts and glare (for each symptom). In comparison 98.2% (56/57), 100% (57/57) and 96.5% (55/57) in the ICB00 group and 98.5% (129/131), 97.7% (129/131) and 97.7% (129/131), in the ZCB00 group reported no difficulty with or did not experience halos, starbursts and glare, respectively.

#### Contrast sensitivity

**Fig. 2:**
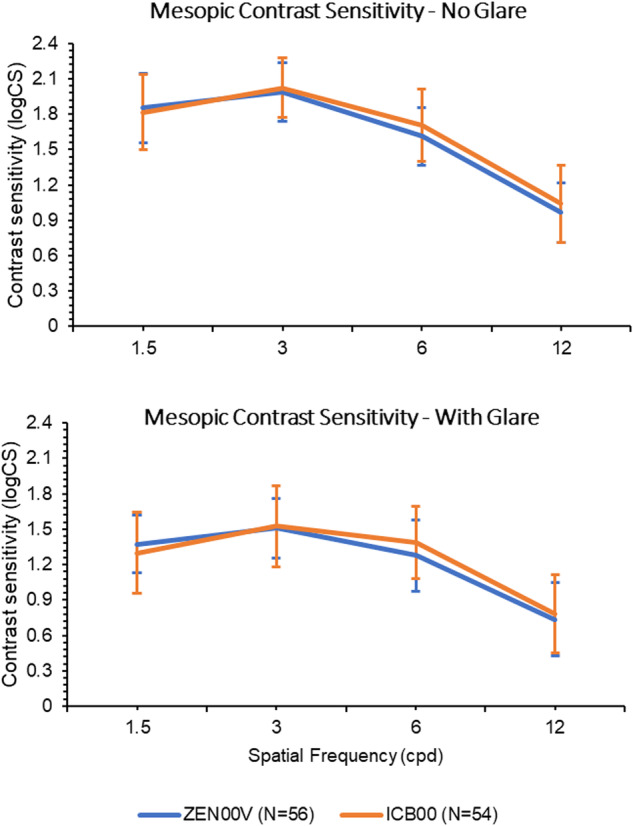
Mean monocular, distance corrected contrast sensitivity under mesopic lighting conditions without glare (top graph), and with glare (bottom graph) for first eyes at 3 months for ZEN00V and ICB00. Error bars represent ±SD.

Figure 2 presents the monocular, corrected distance contrast sensitivity results at 3 months under mesopic conditions with and without glare in both ZEN00V and ICB00 groups. The mean values for contrast sensitivity were comparable between the ZEN00V and ICB00 IOL groups, with differences between the IOL groups falling within 0.11 log units for all measured cycles per degree under both conditions for all spatial frequencies.

### Range of vision

#### Monocular distance corrected intermediate and near visual acuities

The mean (±SD) monocular photopic DCIVA at 66 cm was 0.13 ± 0.08 logMAR (Snellen 20/25) in the ZEN00V group compared to 0.18 ± 0.14 logMAR (Snellen 20/32) in the ICB00 group. The ZCB00 group showed 0.34 ± 0.16 logMAR (Snellen 20/40). In comparison to the ICB00 group ZEN00V group demonstrated a statistically significant improvement in DCIVA of 0.05 logMAR (0.5 lines in Snellen equivalent; *p* = 0.0127).

The median monocular DCIVA at 6 months was 0.13 logMAR (Snellen 20/25) for ZEN00V compared with 0.16 logMAR (Snellen 20/32) for ICB00. Notably, the ZEN00V IOL demonstrated superior and more consistent outcomes, with 81.7% (49/60) achieving 0.20 logMAR (Snellen 20/32) or better monocular DCIVA compared to 60.3% (35/58) in the ICB00 group. The ZCB00 group demonstrated a median monocular DCIVA of 0.34 logMAR, with 18.5% (24/131) achieving 0.20 logMAR (Snellen 20/32) or better.

The mean (±SD) monocular photopic DCNVA at 40 cm was 0.37 ± 0.10 logMAR (Snellen 20/50) in the ZEN00V group and 0.43 ± 0.16 logMAR (Snellen 20/50) in the ICB00 group showing a statistically significant improvement of 0.06 logMAR (0.6 lines in Snellen equivalent; *p* = 0.0137). In comparison, the ZCB00 group showed 0.52 ± 0.19 logMAR (Snellen 20/70).

#### Monocular defocus curve

**Fig. 3:**
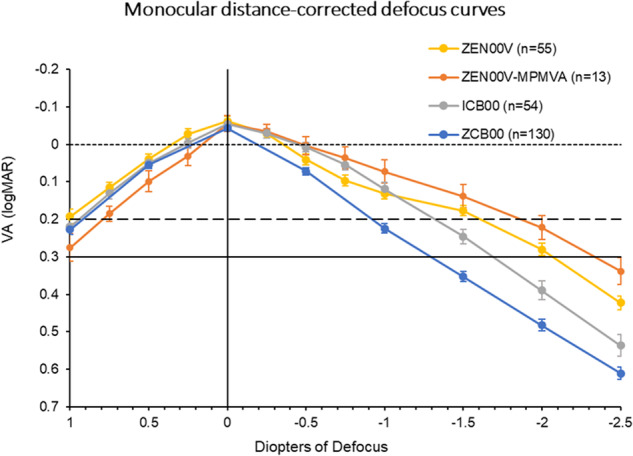
Mean monocular, distance corrected defocus curves at 3 months for ZEN00V and ICB00 ranging from +1.00 D to −2.50 D and at 6 months for ZCB00 (historical control), ranging from +1.00 D to −2.50 D. The defocus curve for ZEN00V from +1.00 D to −2.50 D, obtained with maximum plus refraction (MPMVA) sub-study, is also depicted. Error bars represent ±SE.

Figure 3 illustrates the monocular distance-corrected defocus curves at 3 months for both the ZEN00V and ICB00 groups. The 6-month defocus curve for ZCB00 and for the subgroup of ZEN00V obtained with MPMVA are also displayed. The monocular negative defocus range, where VA of 0.20 logMAR (Snellen 20/32) or better was achieved, extended to −1.6 D for ZEN00V and −1.3 D for ICB00 at 3 months, −0.9 D for ZCB00 at 6 months and −1.9 D for ZEN00V with MPMVA. The ZEN00V IOL outperformed the ICB00 and ZCB00 IOLs by −0.3 D and −0.7 D, respectively, in maintaining VA of 0.20 logMAR (Snellen 20/32) or better, demonstrating an extended range of vision. The ZEN00V IOL with MPMVA outperformed the standard refraction by −0.3 D, underscoring the critical role of achieving maximum plus refraction for assessing the performance of EDF lenses [3]. The ZEN00V IOL with MPMVA surpassed the ICB00 and ZCB00 IOLs by −0.6 D and −1.0 D, respectively, in maintaining VA of 0.20 logMAR (Snellen 20/32) or better.

Stratification by pupil size revealed no discernible pupil dependency of the ZEN00V IOL when compared with ICB00 IOL for pupils larger than 2.5 mm. The sample size for pupils smaller than 2.5 mm was insufficient for meaningful between-group comparisons.

#### Refractive outcomes

At 6 months, ZEN00V exhibited a mean manifest refraction spherical equivalent (MRSE) of 0.083 ± 0.32 D and a mean refractive cylinder of 0.44 ± 0.35 D, compared to −0.20 ± 0.41 D and 0.39 ± 0.26 D for ICB00, respectively. 91.7% (55/60) of ZEN00V and 87.9% (51/58) of ICB00 first eyes achieved absolute MRSE within ±0.50 D of emmetropia. None of the ZEN00V eyes had an MRSE exceeding ±1.00 D, compared to 1.7% (1/58) of ICB00 eyes. For refractive cylinder, none of the ICB00 eyes exceeded ±1.00 D, compared to 6.7% (4/60) of ZEN00V eyes (all within 1.00 to 1.50 D). Historical ZCB00 data at 6 months, showed a mean MRSE of −0.18 ± 0.43 D and a mean refractive cylinder of 0.28 ± 0.33 D. 89.3% (117/131) achieved an absolute MRSE within ±0.50 D, 99.2% (130/131) within ±1.00 D, and 87.0% (114/131) achieved an absolute cylinder within ±0.50 D, and 98.5% (129/131) within ±1.00 D.

#### Serious adverse events and device-related adverse events

Medical complications/adverse events were similar between both the EDF and enhanced monofocal IOL groups and compared favourably to the ISO 11979-9 SPE rates [20]. Five ocular serious and/or device-related adverse events (SAEs/ADEs) occurred in the study with three events occurring in the ZEN00V group and two in the ICB00 group. Two SAEs required secondary surgical interventions (SSIs) for treatment. None of the events were unanticipated.

One ZEN00V AE was a bilateral device-related adverse event of undesirable optic phenomenon. Two non-device-related SAEs in ZEN00V eyes occurred: vitreous strand in a second eye which was treated with Nd:YAG vitreolysis, and a report of toxic anterior segment syndrome (TASS) in a first eye which was successfully treated. Both events resolved without sequelae.

One ICB00 second eye experienced a non-device-related SAE of cystoid macular oedema and an ICB00 first eye experienced a possibly device-related SAE of capsular phimosis which was treated with an YAG anterior capsulotomy. Both events resolved without sequelae.

Other medical and lens findings/AEs in this study were typical and within expected ranges for the test and control IOLs.

## Discussion

The study results show that the TECNIS PureSee IOL (ZEN00V) extends the range of vision and provides improved intermediate and near vision while maintaining distance vision comparable to a monofocal IOL. Subjects implanted with the PureSee IOL achieved on average monocular distance-corrected visual acuity of 20/15 for distance and 20/25 for intermediate vision. The control lens in this study, the TECNIS Eyhance (ICB00), is an enhanced monofocal IOL which is designed to slightly increase the depth of focus [3]. In comparison to this control, the PureSee IOL showed superior intermediate vision and non-inferior distance vision. In defocus testing, the PureSee IOL outperformed the enhanced and standard monofocal IOLs by −0.3 D and −0.7 D, respectively, in maintaining VA of 0.20 logMAR or better. Therefore, the PureSee IOL is expected to meet all effectiveness criteria set forth by the ANSI Z80.35-2018 standards.

The results of the sub-study demonstrated the importance of refractive technique to assess the optimal performance characteristics of presbyopia correcting IOLs (Fig. 3). The sub-study using the MPMVA refraction technique increased the negative monocular defocus range of PureSee by 0.3 D, thereby achieving a total of −1.9 D range over which the subjects were able to maintain visual acuity of 0.20 logMAR or better. This would suggest the negative defocus ranges for the PureSee IOL may be greater than that of the enhanced and standard monofocal IOLs by −0.6 D and −1.0 D, respectively. A prior investigation [3] showcased the advantage of employing maximum refractive technique on visual performance with TECNIS Eyhance. Therefore, adopting the approach of refracting patients with an emphasis on maximum plus for best vision technique is instrumental in maximizing both distance and intermediate vision for extended depth of focus IOLs.

Current EDF and multifocal IOLs may be associated with decreased contrast sensitivity [4, 6, 7, 9, 14]. In this study, the PureSee IOL demonstrated no compromise in mean monocular contrast sensitivity under mesopic conditions. Since there was no compromise in contrast sensitivity with that enhanced monofocal IOL compared to a standard monofocal [3], PureSee IOL contrast sensitivity is expected to be on par with any aspheric monofocal IOL, demonstrating a clinically beneficial technological advancement in the EDF category.

Current EDF and multifocal IOLs are also known to have more noticeable dysphotopsias than monofocal IOLs [4, 7, 16]. While it is difficult to directly compare patient reported outcomes across studies due to the wide variety of questionnaires used, newer lenses have tended to show decreased incidence of severe dysphotopsias compared with previous generation of multifocals [6]. In one study for the TECNIS Symfony IOL, the highest incidence rate for the two worst categories of bother (out of five) for glare, halos and starbursts was 17.0%, compared to 6.8% for the monofocal control [21]. In another recent study for the Vivity EDF IOL, the highest incidence rate for similar parameters was 8.3% [22]. In this study, the direct assessment of dysphotopsias using a validated patient-reported outcomes questionnaire showed that the PureSee IOL provided a dysphotopsia profile comparable to that of the monofocal control and likely on par with current refractive EDF IOLs. The highest incidence rate of very/extremely bothersome glare, halos and starbursts was 3.4%. Most of the patients implanted with this new refractive EDF IOL did not perceive or were not bothered by halos, starburst and glare.

The strength of this study is the randomized, controlled trial design, which eliminates systematic bias. In addition, both evaluators and subjects were masked to IOL type, further minimizing bias. Results from a standard monofocal IOL were presented as historical data to enhance the understanding of the effectiveness and safety of the ZEN00V EDF IOL. However, future trials could include an additional standard monofocal IOL arm for direct comparison between the IOLs.

In conclusion, the TECNIS PureSee IOL provides improved intermediate and near vision while maintaining high-quality distance vision, contrast sensitivity and low rates of bothersome dysphotopsias. Although modern multifocal IOLs are more likely to provide better near vision and therefore, more spectacle independence, than the EDF IOLs, each individual patient needs to weigh whether that improvement outweighs the adverse effects of multifocal IOLs, with increased rates of dysphotopsias and loss in contrast.

## Summary

### What was known before

- Standard monofocal IOLs often require patients to wear spectacles for reading or other near and intermediate tasks.
- Multifocal IOLs are able to provide good near and decent intermediate vision, but are often associated with dysphotopsias (e.g., halos) and lower contrast sensitivity than monofocal IOLs.

### What this study adds

- The TECNIS PureSee IOL is a novel and effective option for cataract surgery patients, providing greater depth of focus with superior intermediate and near vision than an enhanced monofocal IOL and excellent distance vision, contrast sensitivity, and low levels of visual symptoms similar to a monofocal IOL.

## Data Availability

The authors do not intend to share individual deidentified participant data. A summarized report with endpoints data tables based on statistical plan and analysis may be requested directly from the corresponding author for consideration. Access to anonymized data may be granted following review. Content with granted access will be available through email or other appropriate formats and for 3 months, upon review and consideration.

## References

[1] Synek S. The latest generation of intraocular lenses, the problem of the eye refraction after cataract surgery. Coll Antropol. 2013;37:217–21. 23837247

[2] Khandelwal SS, Jun JJ, Mak S, Booth MS, Shekelle PG. Effectiveness of multifocal and monofocal intraocular lenses for cataract surgery and lens replacement: a systematic review and meta-analysis. Graefes Arch Clin Exp Ophthalmol. 2019;257:863–75. 30627791 10.1007/s00417-018-04218-6

[3] Auffarth GU, Gerl M, Tsai L, Janakiraman DP, Jackson B, Alarcon A, et al. Clinical evaluation of a new monofocal IOL with enhanced intermediate function in patients with cataract. J Cataract Refract Surg. 2021;47:184–91. 32932369 10.1097/j.jcrs.0000000000000399

[4] Schallhorn JM. Multifocal and extended depth of focus intraocular lenses: a comparison of data from the United States food and drug administration premarket approval trials. J Refract Surg. 2021;37:98–104. 33577695 10.3928/1081597X-20201111-02

[5] Barnett BP. FOCUSED (Femtosecond Optimized Continuous Uncorrected Sight with EDOF and Diffractive Multifocal IOLs) - A Review. Curr Opin Ophthalmol. 2021;32:3–12. 33122490 10.1097/ICU.0000000000000723

[6] Rampat R, Gatinel D. Multifocal and extended depth-of-focus intraocular lenses in 2020. Ophthalmology. 2021;128:e164–e185. 10.1016/j.ophtha.2020.09.026 32980397

[7] de Silva SR, Evans JR, Kirthi V, Ziaei M, Leyland M. Multifocal versus monofocal intraocular lenses after cataract extraction. Cochrane Database Syst Rev. 2016;12:CD003169. 27943250 10.1002/14651858.CD003169.pub4 PMC6463930

[8] Chang DH, Janakiraman DP, Smith PJ, Buteyn A, Domingo J, Jones JJ, et al. Visual outcomes and safety of an extended depth-of-focus intraocular lens: results of a pivotal clinical trial. J Cataract Refract Surg. 2022;48:288–97. 34269326 10.1097/j.jcrs.0000000000000747 PMC8865208

[9] Kohnen T, Suryakumar R. Extended depth-of-focus technology in intraocular lenses. J Cataract Refract Surg. 2020;46:298–304. 32126045 10.1097/j.jcrs.0000000000000109

[10] Auffarth GU, Moraru O, Munteanu M, Tognetto D, Bordin P, Belucci R, et al. European, multicenter, prospective, non-comparative clinical evaluation of an extended depth of focus intraocular lens. J Refract Surg. 2020;36:426–34. 32644164 10.3928/1081597X-20200603-01

[11] Campos N, Loureiro T, Rodrigues-Barros S, Rita Carreira A, Moraes F, Carreira P, et al. Preliminary clinical outcomes of a new enhanced depth of focus intraocular lens. Clin Ophthalmol. 2021;15:4801–7. 34992340 10.2147/OPTH.S344379 PMC8714966

[12] Ang RE. Visual performance of a small-aperture intraocular lens: first comparison of results after contralateral and bilateral implantation. J Refract Surg. 2020;36:12–9. 31917846 10.3928/1081597X-20191114-01

[13] Dick HB, Elling M, Schultz T. Binocular and monocular implantation of small-aperture intraocular lenses in cataract surgery. J Refract Surg. 2018;34:629–31. 30199568 10.3928/1081597X-20180716-02

[14] McCabe C, Berdahl J, Reiser H, Newsom TH, Cibik L, Koch D, et al. Clinical outcomes in a U.S. registration study of a new EDOF intraocular lens with a nondiffractive design. J Cataract Refract Surg. 2022;48:1297–304. 35616507 10.1097/j.jcrs.0000000000000978 PMC9622364

[15] Mencucci R, Favuzza E, Caporossi O, Savastano A, Rizzo S. Comparative analysis of visual outcomes, reading skills, contrast sensitivity, and patient satisfaction with two models of trifocal diffractive intraocular lenses and an extended range of vision intraocular lens. Graefes Arch Clin Exp Ophthalmol. 2018;256:1913–22. 29980919 10.1007/s00417-018-4052-3

[16] Arrigo A, Gambaro G, Fasce F, Aragona E, Figini I, Bandello F. Extended depth-of-focus (EDOF) AcrySof(R) IQ Vivity(R) intraocular lens implant: a real-life experience. Graefes Arch Clin Exp Ophthalmol. 2021;259:2717–22. 34050809 10.1007/s00417-021-05245-6

[17] Alarcon A, Canovas C, Koopman B, Weeber H, Auffarth GU, Piers PA. Enhancing the intermediate vision of monofocal intraocular lenses using a higher order aspheric optic. J Refract Surg. 2020;36:520–7. 32785725 10.3928/1081597X-20200612-01

[18] American National Standards Institute. ANSI Z80.35-2018. Ophthlamics – Extended Depth of Focus Intraocular Lenses. Alexandria, VA: ANSI; 2018. Available online: https://webstore.ansi.org/standards/vc%20(asc%20z80)/ansiz80352018r2023.

[19] FDA SSED Tecnis Synergy IOLs. Available online: https://www.accessdata.fda.gov/scripts/cdrh/cfdocs/cfpma/pma.cfm?id=P980040S124 (2021).

[20] International Organization for Standardization. ISO 11979-7:2018(en). Ophthalmic Implants — Intraocular lenses. Part 7: Clinical investigations of intraocular lenses for the correction of aphakia. Geneva: ISO; 2018. Available online: https://www.iso.org/standard/69038.html#:~:text=ISO%2011979%2D7%3A2018%20specifies,in%20order%20to%20correct%20aphakia.

[21] FDA SSED Tecnis Symfony IOLs. Available online: https://www.accessdata.fda.gov/scripts/cdrh/cfdocs/cfpma/pma.cfm?id=P980040S065 (2016).

[22] Pantanelli SM, O’Rourke T, Bolognia O, Scruggs K, Longenecker A, Lehman E. Vision and patient-reported outcomes with nondiffractive EDOF or neutral aspheric monofocal intraocular lenses. J Cataract Refract Surg. 2023;49:360–6. 36728998 10.1097/j.jcrs.0000000000001123 PMC10050137

