# Correction: Quality of vision clinical outcomes for a new fully-refractive extended depth of focus intraocular lens

**DOI:** 10.1038/s41433-024-03421-6

**Published:** 2024-11-05

**Authors:** Dean Corbett, Daniel Black, Timothy V. Roberts, Brendan Cronin, David Gunn, Chandra Bala, Patrick Versace, Linda Tsai, Eleni Papadatou, Aixa Alarcon, Srividhya Vilupuru

**Affiliations:** 1 Auckland Eye, Auckland, New Zealand; 2 Sunshine Eye Clinic, Birtinya, QLD Australia; 3 Vision Eye Institute, Sydney, NSW Australia; 4 The University of Sydney, Sydney Medical School, Faculty of Medicine and Health, Sydney, NSW Australia; 5 Queensland Eye Institute Foundation, South Brisbane, QLD Australia; 6 personalEYES, Parramatta, NSW Australia; 7 Dr Versace, SMS healthcare, University of New South Wales, Sydney, NSW Australia; 8 Johnson and Johnson MedTech, Irvine, CA USA; 9 Johnson and Johnson MedTech, Groningen, The Netherlands

**Keywords:** Outcomes research, Lens diseases

Correction to: *Eye* 10.1038/s41433-024-03039-8, published online 05 April 2024

The second sentence in the section ‘SUBJECTS/METHODS’ of the Abstract has been corrected from: “Subjects were bilaterally implanted with the EDF test (Model ZEN00V, *N* = 60) or an enhanced monofocal control (Model ICB00, *N* = 57) IOL.” to: “Subjects were bilaterally implanted with the EDF test (Model ZEN00V, TECNIS PureSee™ IOL, *N* = 60) or an enhanced monofocal control (Model ICB00, TECNIS Eyhance™ IOL, *N* = 57) IOL.” The original article has been corrected.

